# *Ficus hispida* Bark Extract Prevents Nociception, Inflammation, and CNS Stimulation in Experimental Animal Model

**DOI:** 10.1155/2017/7390359

**Published:** 2017-04-12

**Authors:** Md. Sariful Islam Howlader, Md. Afjalus Siraj, Shubhra Kanti Dey, Arpona Hira, Arif Ahmed, Md. Hemayet Hossain

**Affiliations:** ^1^Pharmacy Discipline, Life Science School, Khulna University, Khulna 9208, Bangladesh; ^2^Department of Pharmaceutical Sciences, Daniel K. Inouye College of Pharmacy, University of Hawaii at Hilo, Hilo, HI 96720, USA; ^3^BCSIR Laboratories, Bangladesh Council of Scientific and Industrial Research (BCSIR), Dhaka 1205, Bangladesh

## Abstract

*Background. Ficus hispida* is traditionally used in the ailment of pain, inflammation, and neurological disorders. The present study set out to evaluate the in vivo antinociceptive, anti-inflammatory, and sedative activity of the ethanol extract of* Ficus hispida* bark (EFHB).* Methods.* The antinociceptive activity of EFHB was evaluated by using acetic acid induced writhing, formalin, hot plate, and tail immersion methods in Swiss albino mice. Its anti-inflammatory activity was assessed by using carrageenan and histamine induced rat paw oedema test in Wister rats. The central stimulating activity was studied by using pentobarbital induced hypnosis, hole cross, and open field tests in Swiss albino mice.* Results.* EFHB demonstrated antinociceptive activity both centrally and peripherally. It showed 62.24% of writhing inhibition. It significantly inhibited licking responses in early (59.29%) and late phase (71.61%). It increased the reaction time to the thermal stimulus in both hot plate and tail immersion. It inhibited the inflammation to the extent of 59.49%. A substantial increase in duration of sleep up to 60.80 min and decrease of locomotion up to 21.70 at 400 mg/kg were also observed.* Conclusion.* We found significant dose dependent antinociceptive, anti-inflammatory, and sedative properties of EFHB in experimental animal models.

## 1. Introduction

*Ficus hispida* (Linn.) (Moraceae) is a medium sized tree generally known as Dumoor in Bangladesh. It is a popular plant which is widely distributed throughout subcontinent including India and Bangladesh [1]. Recent publications revealed the presence of several new alkaloids in the twigs, steam-bark, and the leaves of the* F. hispida*. Twigs contain ficushispimines A and B (pyrrolidine alkaloid), ficushispimine C (*ω*-(dimethylamino)caprophenone alkaloid), and ficushispidine (indolizidine alkaloid) whereas hispiloscine (phenanthroindolizidine alkaloid) was isolated from the stem-bark and leaves [2, 3]. Moreover, the barks of* F. hispida* contain *β*-amyrin acetate, N-triacontanyl acetate, lupeol acetate, and 10-keto-tetracosyl arachidate [4]. Pharmacological properties of the whole plant, fruit, root, and leaves of* F. hispida* have already been reported. The whole plant has astringent, antidysenteric, antipsoriasis, antianemic, and antihemorrhagic properties [5, 6]. The fruit is edible and acts as a coolant and tonic. A mixture of honey and its juice is a good antihemorrhagic [7]. The roots and leaves are reported for their antidiarrhoeal [8], antidiabetic [9], antibacterial [10], hepatoprotective [11], antioxidant [12], and cardioprotective [13] properties. The leaves and fruits have been also reported for their analgesic activity in acetic acid-induced writhing model in mice [14].

Traditionally in Bangladesh,* F. hispida* bark is used against pain, inflammation, and neurological disorders such as epilepsy and depression. However, very limited pharmacological investigation was carried out on* F. hispida* bark till date. Therefore, the present study was designed to explore the in vivo antinociceptive, anti-inflammatory, and sedative activity of* F. hispida* bark.

## 2. Materials and Methods

### 2.1. Plant Collection and Extraction

*F. hispida* barks were collected from the fresh plants from Chittagong district, Bangladesh. The samples were deposited at Bangladesh National Herbarium (BNH) and identified by the experts. A voucher specimen (DACB: 35921) was submitted there for further reference. The barks were shade dried and crushed into powder with the help of Capacitor Start Motor, China. Five hundred grams (500 g) of bark powder was produced which was transferred into a clean glass vessel and soaked in 1.5 L of ethanol. The glass vessel was airtight and saved for 7 days at room temperature with random shaking. After 1 week, the mixture was filtered initially by a piece of sterilized cotton material followed by Whatman 24 cm filter paper (Bibby RE200, Sterilin Ltd., UK) and revised several times to get a very clear filtrate. After that, the ethanol was recovered by using rotary vacuum evaporator (R-205, Buchi, Switzerland) at reduced pressure. The ethanol extract was then dried with the help of SpeedVac (RVT4104, Thermo Scientific). It yielded a 66 g of gummy concentrate (13.20%) crude ethanol extract.

### 2.2. Chemicals and Drugs

Diclofenac sodium, morphine sulphate, naloxone, carrageenan, histamine phosphate, indomethacin, gallic acid, quercetin, pentobarbitone, and Folin-Ciocalteu phenol reagent were collected from Sigma Chemical Co. (St. Louis, MO, USA). Ethanol and formalin were purchased from E. Merck, Germany. Analytical grade Tween 80, sodium carbonate, aluminum chloride, and potassium acetate were bought from E. Merck, India, Ltd.

### 2.3. Test Animals

For laboratory animal studies, Swiss albino mice (20–25 g) and Wister rats (175–202 g) of both sexes were used. These animals were collected from the animal research branch of International Center for Diarrheal Disease Research, Bangladesh (ICDDR, B). They were kept under standard laboratory environments maintained at 25 ± 2°C and under 12/12 h light/dark cycle and fed with standard diet and water ad libitum during adaptation period. The animals were put away from any food/water overnight before the experiments. During the experimental period, these animals were treated according to the “Ethical Principles and Guidelines for Scientific Experiments on Animals (1995)” formulated by the Swiss Academy of Medical Sciences and the Swiss Academy of Sciences. The experimental procedure was approved by the Bangladesh Council of Scientific and Industrial Research (BCSIR) ethics committee (BCSIR/IAEC/08/11-12).

### 2.4. Phytochemical Screening

Phytochemical screening of EFHB was carried out by using standard quantitative procedures to investigate the presence of alkaloid, steroid, reducing sugar, saponin, tannin, and flavonoid [15, 16].

### 2.5. Determination of Total Phenolic Content

Total phenolic content of EFHB was determined by modified Folin-Ciocalteu method [17]. 1 mg/ml of EFHB was added with the mixture of Folin-Ciocalteu reagent (5 ml) and 75 g/l of Na-carbonate solution (4 ml). The combination was kept at 37°C for half an hour and the absorbance was measured by UV spectrophotometer at 765 nm (analytikjena, Model 205, Germany). Finally, total phenolic content was calculated (mg of gallic acid/g of dry extract) by using the standard gallic acid calibration curve equation: *y* = 6.2548*x* − 0.0925 and *R*^2^ = 0.9962.

### 2.6. Determination of Total Flavonoid Content

Total flavonoid content of EFHB was determined by AlCl_3_ colorimetric method [18]. EFHB was mixed properly with the combination of methanol (1.5 ml), 10% aluminum chloride (0.1 ml), 1 M potassium acetate (0.1 ml), and distilled water (2.8 ml). It was then kept at 37°C for half an hour. The absorbance was measured by UV spectrophotometer at 415 nm (analytikjena, Model 205, Germany). Finally, total flavonoids content was calculated (mg/g of quercetin equivalent) by using the quercetin calibration curve equation: *y* = 4.7385*x* + 0.0355 and *R*^2^ = 0.9993.

### 2.7. Acute Toxicity Test

Acute toxicity test of EFHB was investigated in order to determine the dose(s) to be used in different tests in the laboratory animals. Rats were kept in fasting condition for 16 h. 500, 1000, 2000, 3000, and 4000 mg/kg EFHB dose were orally administered to fasting rats. After that, they were allowed free access to food and water and all the rats were under careful observation over a period of 72 h and the number of deaths within this period was recorded [19].

### 2.8. Antinociceptive Tests

#### 2.8.1. Acetic Acid-Induced Writhing Test

In the acetic acid-induced writhing model in mice, different groups of animals (five mice/group, total twenty-five) were treated with 1% Tween 80 in normal saline (10 ml/kg, p.o.), EFHB (100, 200, and 400 mg/kg, p.o.), and diclofenac sodium (25 mg/kg, i.p.) 30 min before intraperitoneal injection of 0.7% acetic acid. After an interval of 5 min, writhing (constriction of the abdomen, turning of trunk, and extension of hind legs) was observed for 10 min [20, 21].

#### 2.8.2. Formalin Test

In the formalin-induced paw licking test in mice, overnight fasted animals (five mice/group, total thirty) were treated with 1% Tween 80 in normal saline (10 ml/kg, p.o.), EFHB (100, 200, and 400 mg/kg, p.o.), morphine (5 mg/kg, s.c.), and diclofenac-Na (25 mg/kg, s.c.). Formalin (20 *μ*l of 1% solution) was injected subcutaneously into the right hind paw of each mouse after 60 min in oral administered group and after 30 min in subcutaneous group. Licking or biting of the treated paw was recorded as nociceptive response for 0–5 min in the early phase and for 15–30 min in the late phase after formalin injection [22, 23].

#### 2.8.3. Hot Plate Test

In this assay, mice were placed on a heated (50 ± 0.05°C) metal plate and the time elapsed until the appearance of reactions (lifting or licking of the paws) to the thermal stimulus was recorded as an index of nociception [24, 25]. A cut-off period of 30 s was imposed to avoid tissue damage to the paws. Twenty-five mice were divided into five equal groups (5 mice/group), which were pretreated with 1% Tween 80 in normal saline (10 ml/kg, p.o.), EFHB (100, 200, and 400 mg/kg, p.o.), and morphine (5 mg/kg, s.c). The response time was noted at different time intervals, that is, 0, 30, 60, 90, and 120 min. In another set of experiments (total 25 mice; 5 mice/group), an opioid nonselective antagonist naloxone (2 mg/kg) was injected (i.p.) 15 min prior to the administration of either morphine (10 mg/kg, s.c.) or EFHB (100, 200, and 400 mg/kg, p.o.). Antinociceptive activity was expressed as the increase in latency time to thermal stimulus with respect to control.

#### 2.8.4. Tail Immersion Test

In this experiments, twenty-five mice were homogeneously divided into five different groups where mice tails (2 cm) pretreated with 1% Tween-80 in normal saline (10 ml/kg, p.o.), EFHB (100, 200, and 400 mg/kg, p.o.), and morphine (5 mg/kg, s.c.) were immersed in warm water (55 ± 1°C). The latency between tail submersion and deflection of the tail was recorded and the pretreatment latency was recorded at 30, 60, 90, and 120 min. This latency period was taken as the index of antinociception. Moreover, an opioid nonselective antagonist naloxone (2 mg/kg) was injected (i.p.) in 25 other mice (5 mice/group) in 15 min prior to the administration of either morphine (10 mg/kg, s.c.) or EFHB and observed as explained above [26].

### 2.9. Anti-Inflammatory Tests

#### 2.9.1. Carrageenan-Induced Oedema Test

Carrageenan-induced rat hind paw oedema was used as the animal model of acute inflammation. In this experiment, twenty-five rats divided into five groups (five rats/group) were treated with 1% Tween 80 in normal saline (10 ml/kg, p.o.); EFHB (100, 200, and 400 mg/kg, p.o.); and indomethacin (10 mg/kg, p.o.). Acute inflammation was induced in groups by subplantar injection of 0.1 ml of carrageenan (1% suspension in Tween 80) in the right paw of rats 1 hour after all of the oral administration. The paw oedema volume was measured with micrometer screw gauze at 1, 2, 3, 4, and 5 h after the administration of the tested materials [27, 28].

#### 2.9.2. Histamine-Induced Oedema Test

In this experiment, after dividing twenty-five rats into five groups (five rats/group), they were treated with 1% Tween 80 in normal saline (10 ml/kg, p.o.), EFHB (100, 200, and 400 mg/kg, p.o.), and indomethacin (10 mg/kg, p.o.). Acute inflammation was induced by subplantar injection of 0.1 ml of histamine with 1% suspension in Tween-80 in the right hind paw of the rats 30 min after the oral administration of the tested materials. The paw volume was measured with micrometer screw gauze at 1, 2, 3, 4, and 5 h after the administration of the tested materials [29].

### 2.10. Neuropharmacological Tests

#### 2.10.1. Pentobarbital Induced Hypnosis

In pentobarbital induced hypnosis test, twenty-five mice divided into five groups (five rats/group) were treated with 1% Tween 80 in normal saline (10 ml/kg), EFHB (100, 200, and 400 mg/kg), and diazepam (1 mg/kg). Each mouse was placed in a quadrangular observation box (36 × 36 cm^2^). After 30 min, pentobarbitone (40 mg/kg) was given to mice to induce sleep. The total sleeping time was monitored for all experimental groups. Mice were observed for the duration of sleep (i.e., time difference between the loss and recovery of reflex) and the latent period (i.e., time difference between pentobarbitone administration and loss of reflex) [30].

#### 2.10.2. Open Field Test

Open field experimental method is routinely used to evaluate emotional and locomotors activities in rodents [31]. A specially designed experimental box was used for this test which had a series of alternatively colored black and white square floor. Five groups containing five mice/group (total twenty-five mice) were treated with 1% Tween 80 in normal saline (10 ml/kg), EFHB (100, 200, and 400 mg/kg), and diazepam (1 mg/kg). After the treatment, the number of squares traveled by the mice was monitored for 3 min. Percentage inhibition of movements was determined by using the following formula:

(1) $$\begin{array}{c} \text{\% movement inhibition}=\frac{\left\{\text{Mean number of movement (control)}-\text{mean number of movement (test)}\right\}}{\text{Mean number of movements (control)}}\times 100. \end{array}$$

#### 2.10.3. Hole Cross Test

Hole cross experimental method was run in a cage (*L* × *W* × *H* = 30 × 20 × 14 cm) containing a fix steel partition in the middle [32]. A 3 cm diameter hole was made at a height of 7.5 cm in the center of the cage. 25 mice were divided into five groups equally. The different groups of animals were administered with 1% Tween 80 in normal saline (10 ml/kg), EFHB (100, 200, and 400 mg/kg), and diazepam (1 mg/kg). Pain stimulus was produced by placing the animals on hot plate maintained at the temperature of 55 ± 0.5°C. Paw licking or jumping off the plate was considered as response to pain stimulus. Reaction time for each group was recorded at 0, 30, 60, 90, and 120 min during the observation period. The number of movements of mice through the hole from one chamber to another was counted after oral administration of tested materials. Percentage inhibition of movements was determined by using (1).

### 2.11. Statistical Analysis

All of the experimental results are reported as mean ± SEM. Statistical analysis was performed using one-way analysis of variance (ANOVA) followed by Dunnett's test [33] using SPSS 11.5 software. Differences between groups were considered significant at a level of *p* < 0.05.

## 3. Results

### 3.1. Antinociceptive Tests

#### 3.1.1. Acetic Acid-Induced Writhing in Mice

At 100, 200, and 400 mg/kg EFHB showed 37.76, 50.35, and 62.24 percentage of writhing episode inhibition, respectively. Standard drug diclofenac sodium (25 mg/kg) showed 75.52%  (*p* < 0.001) reduction (Table 2).

#### 3.1.2. Formalin Test

EFHB demonstrated significant inhibition of licking responses in early (44.57%, 50.36%, and 59.29%) and late phase (51.64%, 60.17%, and 71.61%) at the doses of 100, 200, and 400 mg/kg, respectively. Standard morphine produced marked inhibition in both the early phase (70.76%) and late phase (99.13%) whereas diclofenac sodium produced inhibition of licking responses (96.32%) in the late phase only (Table 3).

#### 3.1.3. Hot Plate Test

EFHB significantly increased the reaction time to the thermal stimulus in a dose dependent manner (3.91, 3.97, and 5.13 s after 120 min at 100, 200, and 400 mg/kg, resp.) which was comparable to the standard drug morphine (5.27 s after 120 min). Naloxone produced a substantial antagonistic effect on the antinociceptive activity of both EFHB and morphine (Table 4).

#### 3.1.4. Tail Immersion Test

EFHB substantially increased the latency period to hot-water induced thermal stimuli (3.71, 4.52, and 5.26 s after 120 min at 100, 200, and 400 mg/kg dose) whereas standard drug morphine at 5 mg/kg showed 3.74 s latency period after 120 min. Naloxone counteracts the antinociceptive activity of both EFHB and morphine (Table 5).

### 3.2. Anti-Inflammatory Tests

#### 3.2.1. Carrageenan-Induced Paw Oedema

EFHB showed a significant reduction in the carrageenan-induced paw volume after 5 h as compared with the control group. EFHB (200 and 400 mg/kg) inhibited the inflammation to the extent of 44.94% and 59.49% while the reference drug, indomethacin, reduced the inflammation by 67.72%  (Table 6).

#### 3.2.2. Histamine-Induced Paw Oedema

At the higher doses (200 and 400 mg/kg), EFHB inhibited the inflammation to the extent of 46.43% and 60.12% which was statistically significant (*p* < 0.05; *p* < 0.01). Indomethacin reduced the inflammation by 69.64%  (Table 7).

### 3.3. Neuropharmacological Tests

#### 3.3.1. Pentobarbital Induced Hypnosis Test

A substantial increase in duration of sleep was observed in the dosages of 200 and 400 mg/kg (53.70 ± 1.77 and 60.80 ± 2.01 min, resp., as compared to 73.50 ± 2.20 min in the control group at *p* < 0.001) in pentobarbital induced hypnosis test. A notable decrease in onset of action was also showed at the doses of 200 and 400 mg/kg as compared to the control group. The results were dose dependent and statistically significant (*p* < 0.001 and *p* < 0.01) (Table 8).

#### 3.3.2. Open Field Test

EFHB showed a noticeable decrease in locomotion in the test animals with increasing time. At 240 min, the number of movements was gradually decreased up to 37.50 (*p* < 0.01), 30.40 (*p* < 0.01), and 21.70 (*p* < 0.001) for the dose of 100, 200, and 400 mg/kg, respectively, whereas movement decreased up to 5.90 (*p* < 0.001) in case of standard drug diazepam (Table 9).

#### 3.3.3. Hole Cross Test

The EFHB displayed suppression of motor activity and exploratory behavior in the test animal. The locomotor activity lowering effect was evident from the 2nd observation period (30 min) and continued up to 7th observation period (240 min). At 240 min, number of holes crossed was gradually decreased up to 6.10 (*p* < 0.01), 5.20 (*p* < 0.01), and 4.30 (*p* < 0.001) for the dose of 100, 200, and 400 mg/kg, respectively. The standard drug, diazepam, decreased the number of holes crossed throughout the observation period (Table 10).

## 4. Discussion

We did not observe any mortality up to the dose of 4 g/kg b.w. of EFHB (p.o.) in the test animals which ensured the safety of the experimental dose.

Acetic acid generally produces pain by increasing the level of PGE_2_ and PGF_2*α*_. It also induces sympathetic nervous system mediators, which were naturally observed at higher level at first 30 min of injection [34]. It excites pain in nerve endings and activates the production of noxious substances within the peritoneum which generates the writhing response. EFHB exhibited significant dose dependent writhing reduction. This result indicated the peripheral antinociceptive effect of EFHB which may occur due to the inhibition of the synthesis or/and action of prostaglandin. Moreover, as the acetic acid mimics the inflammatory local pain, it may also suggest the probable association of EFHB with the peripheral inhibition of bradykinins and prostanoids [35]. This may be due to the presence of *β*-amyrin acetate which is previously reported for its antinociceptive activity [36].

Formalin test was performed since the results of the writhing test alone could not ascertain the source of antinociception. It evaluates antinociceptive property in two distinct phases. In the early/first phase, neurogenic pain is induced by direct chemical stimulation of the sensory afferent fibers; particularly c-fibers. The involvement of substance P and bradykinin has also been reported. Pain is induced by prostaglandins, histamine, bradykinin, and serotonin in the late phase [37]. Drugs that act on the central nervous system inhibit both phases while peripherally acting drugs inhibit the late phase only [38]. EFHB suppressed the licking time in both phases and appeared effective on both tonic inflammatory and central pain.

The hot plate and tail immersion tests are widely used for assessing central antinociceptive activity. They are distinguished by their tendency to respond to the pain stimuli, as the hot plate demonstrates supraspinal reflex mediated by *μ*_1_ and *μ*_2_ opioid receptors while the tail immersion monitors a spinal reflex involving *μ*_2_ and *δ* opioid receptors [39, 40]. Opioid agents exhibit their analgesic effects both via supraspinal (*κ*_3_, *μ*_1_, *δ*_1_, and *σ*_2_) and spinal (*μ*_2_, *δ*_2_, and *κ*_1_) receptors [39]. In our experiments, EFHB exhibited significant dose dependent but lesser antinociceptive activity than morphine. To verify possible centrally acting antinociceptive mechanism of EFHB, the inhibitory effect of naloxone was carried out. *μ* opioid receptor is the major target site for morphine, while naloxone reversed the antinociceptive effect of morphine. Likewise, naloxone also reversed the analgesic response of EFHB against thermal stimuli. This can be explained by the presence of *β*-amyrin acetate which is responsible for analgesic effect by vanilloid receptor (TRPV_1_) [41] and also by reducing neuropathic hyperalgesia produced by the direct activation of the cannabinoid receptors [42].

The inflammatory response induced by carrageenan is characterized by a biphasic response with marked oedema formation due to the rapid production of inflammatory mediators. These mediators are subsequently sustained by the release of prostaglandins and nitric oxide which is produced by cyclooxygenase (COX-2) and nitric oxide synthase (iNOS), respectively [43]. EFHB was effective in reducing the oedematogenic response between the 3rd and 5th h after carrageenan administration signifying that EFHB is able to inhibit one or more intracellular inflammatory signaling pathways. On the other hand, histamine increases the vascular permeability and is known as an important inflammatory mediator [42]. When histamine is subcutaneously injected, it forms a wheal around the injected place due to increase of vascular permeability of the host capillary venules. Substances that antagonize this activity reduce the area. Since EFHB effectively suppressed the oedema in a dose dependent manner, it can be suggested that EFHB exhibited anti-inflammatory actions by inhibiting the synthesis or action of histamine. The bark of* F. hispida* contains *β*-amyrin acetate which is reported for its anti-inflammatory activity by decreasing IL-1*β*, TNF-*α*, KC, and IL-6 levels and by inhibiting expression of NF-*κ*B and COX-2 [44]. It also contains lupeol acetate, which produces anti-inflammatory activity by regulating TNF-*α* and IL-2 [45]. Moreover, plant derived phenolic and flavonoids often exhibit antioxidant potential [46]. Thus they protect the cells from ROS and generate anti-inflammatory activity [47]. In this study, EFHB showed significant amount of phenolic and flavonoid levels and may have been responsible for the potent anti-inflammatory activity recorded (Table 1).

A CNS depressant works either by decreasing the onset or by increasing the duration of sleep or both. EFHB increased the pentobarbitone-induced sedative effect in a dose dependent manner. Barbiturates naturally work on the cerebral cortex to generate their action [48]. EFHB improved sleeping time that can be attributed to its action on the central sleeping mechanism [49]. Moreover, EFHB decreased the locomotor activity which is a parameter of the level of excitability of the CNS [50]. Decrease of locomotor activity is closely related to the depression of the CNS [51]. Sedative-hypnotic drugs generate their bioactivity through GABA_A_ receptor [52]. EFHB may act by potentiating GABAergic inhibition via membrane hyperpolarization. The sedative activity of EFHB can also be correlated with the presence of *β*-amyrin acetate in the* F. hispida* bark. *β*-Amyrin acetate is already reported for its sedative and anxiolytic effects [53]. Furthermore, flavonoids were found to be ligands for the GABA_A_ receptor [54]. Tannin, saponin, and glycoside are reported for their sedative effects [55].

## 5. Conclusion

Present study showed potent antinociceptive, anti-inflammatory, and sedative activities of EFHB. All activities were dose dependent and statistically significant. Presence of *β*-amyrin acetate, lupeol acetate, and phenolic and flavonoid constituents may play an important role in these bioactivities. We are hopeful that these research findings will definitely provide a rationale for further chemical and biological study on EFHB in the near future.

## Figures and Tables

**Table 1:** Total phenolic and flavonoids content of EFHB.

Treatment	Total phenolic content (mg of gallic acid per g of dry extract)	Total flavonoids content (mg of quercetin per g of dry extract)
EFHB	258.37 ± 6.68	144.29 ± 7.89

Here, each value is presented as the mean ± SEM (*n* = 3). EFHB = ethanol extract of *Ficus hispida* barks.

**Table 2:** Effects of EFHB on acetic acid-induced writhing test in mice.

Treatment	Dose (mg/kg, p.o.)	Number of writhes	Inhibition (%)
Vehicle	10 (ml/kg)	14.3 ± 0.79	—
Diclofenac sodium	25	3.5 ± 0.38^*∗∗*^	75.52
EFHB	100	8.9 ± 0.45^*∗*^	37.76
EFHB	200	7.1 ± 0.36^*∗∗*^	50.35
EFHB	400	5.4 ± 0.63^*∗∗*^	62.24

Here, each value is presented as the mean ± SEM (*n* = 5). EFHB = ethanol extract of *Ficus hispida*.

^*∗∗*^Indicating *p* < 0.01 compared with control group (Dunnett's test).

^*∗*^Indicating *p* < 0.001 compared with control group (Dunnett's test).

**Table 3:** Effects of EFHB on formalin-induced paw licking test in mice.

Treatment	Dose (mg/kg)	Licking of the hind paw			
		Early phase (0–5 min)	% inhibition	Late phase (15–30 min)	% inhibition
Vehicle	10 (ml/kg)	98.50 ± 4.27	—	103.20 ± 4.93	—
Morphine	5	28.80 ± 2.92^*∗*^	70.76	0.90 ± 0.11	99.89
Diclofenac sodium	10	62.20 ± 3.85^*∗∗*^	36.85	3.80 ± 0.57^*∗∗*^	96.25
EFHB	100	54.60 ± 3.31^*∗*^	44.46	49.90 ± 2.88^*∗*^	51.64
EFHB	200	48.90 ± 2.54^*∗*^	50.36	41.10 ± 4.17^*∗∗*^	60.17
EFHB	400	40.10 ± 3.22^*∗*^	59.29	29.30 ± 3.92^*∗∗*^	71.61

Here, each value is presented as the mean ± SEM (*n* = 5). EFHB = ethanol extract of *Ficus hispida*.

^*∗∗*^Indicating *p* < 0.01 compared with control group (Dunnett's test).

^*∗*^Indicating *p* < 0.001 compared with control group (Dunnett's test).

**Table 4:** Effects of EFHB on hot plate test in mice.

Treatment	Dose (mg/kg)	Latency period (s) (% MPE)					
		0 min	30 min	45 min	60 min	90 min	120 min
Vehicle	10 (ml/kg)	2.19 ± 0.19	2.40 ± 0.16	2.42 ± 0.23	2.47 ± 0.34	2.39 ± 0.38	2.69 ± 0.45
Morphine	5	2.38 ± 0.21	10.63 ± 0.65 (41.15)^*∗∗*^	10.31 ± 0.57 (39.31)^*∗*^	10.98 ± 0.69 (42.71)^*∗∗*^	9.92 ± 0.54 (34.86)^*∗∗*^	5.27 ± 0.30 (11.08)^*∗∗*^
EFHB	100	2.24 ± 0.22	2.71 ± 0.19 (2.64)^*∗*^	3.45 ± 0.53 (6.78)^*∗*^	3.87 ± 0.75 (9.74)^*∗*^	4.23 ± 0.49 (11.21)^*∗∗*^	3.91 ± 0.56 (9.74)^*∗*^
EFHB	200	2.29 ± 0.23	2.93 ± 1.56 (3.83)^*∗*^	3.71 ± 0.61 (8.23)^*∗*^	4.51 ± 0.60 (12.73)^*∗*^	5.31 ± 0.53 (17.24)^*∗∗*^	3.97 ± 0.60 (9.35)^*∗*^
EFHB	400	2.37 ± 0.19	3.89 ± 0.43 (8.60)^*∗∗*^	5.60 ± 0.59 (18.31)^*∗*^	7.67 ± 0.58 (18.16)^*∗∗*^	6.78 ± 0.54 (19.30)^*∗∗*^	5.13 ± 0.35 (12.26)^*∗*^
NLX	2	2.65 ± 0.35	2.51 ± 0.51	2.65 ± 0.34	2.39 ± 0.26	2.58 ± 0.38	1.77 ± 0.28
NLX + morphine	2 + 5	2.90 ± 0.31	2.59 ± 0.43 (−2.02)^a^	3.92 ± 0.27 (0.74)^a^	2.76 ± 0.27 (−0.72)^a^	2.31 ± 0.22 (−3.64)^a^	2.88 ± 0.26 (−0.63)^a^
NLX + EFHB	2 + 100	2.97 ± 0.32	2.30 ± 0.27 (−3.42)	2.21 ± 0.47 (−4.01)^b^	2.27 ± 0.64 (−1.35)^b^	2.65 ± 0.83 (−1.07)^b^	2.97 ± 0.31 (−0.04)^b^
NLX + EFHB	2 + 200	2.35 ± 0.20	2.19 ± 0.14 (1.11)^c^	2.18 ± 0.56 (−0.33)^c^	2.59 ± 0.62 (1.21)^c^	2.66 ± 0.79 (−0.27)^c^	2.18 ± 0.26 (−0.99)^c^
NLX + EFHB	2 + 400	2.71 ± 0.71	2.87 ± 0.30 (2.07)	3.22 ± 0.44 (3.34)^d^	3.10 ± 0.58 (2.44)^d^	2.91 ± 0.48 (1.28)^d^	2.27 ± 0.18 (−3.49)^d^

Here, each value is presented as the mean ± SEM (*n* = 5). EFHB = ethanol extract of *Ficus hispida*. NLX = naloxone, MPE = Maximum Possible Effect.

^*∗*^Indicating *p* < 0.05 compared with control group (Dunnett's test).

^ ^^*∗∗*^Indicating *p* < 0.01 compared with control group (Dunnett's test).

^a^Indicating *p* < 0.001 compared with morphine group (Bonferroni's test).

^b^Indicating *p* < 0.05 compared with morphine group (Bonferroni's test).

^c^Indicating *p* < 0.05 compared with morphine group (Bonferroni's test).

^d^Indicating *p* < 0.01 compared with morphine group (Bonferroni's test).

**Table 5:** Effects of EFHB on tail immersion plate test in mice.

Treatment	Dose (mg/kg)	Latency period (s) (% MPE)					
		0 min	30 min	45 min	60 min	90 min	120 min
Vehicle	10 (ml/kg)	2.15 ± 0.29	2.19 ± 0.31	2.74 ± 0.29	2.25 ± 0.34	2.33 ± 0.31	2.44 ± 0.27
Morphine	5	2.28 ± 0.15	10.03 ± 0.73 (41.15)^*∗∗*^	12.51 ± 0.62 (42.71)^*∗∗*^	11.82 ± 0.46 (42.71)^*∗∗*^	8.25 ± 0.44 (34.86)^*∗∗*^	3.74 ± 0.2 (11.08)^*∗∗*^
EFHB	100	2.90 ± 0.28	4.75 ± 0.48 (2.64)^*∗*^	6.56 ± 0.31 (9.74)^*∗*^	5.86 ± 0.43 (9.74)^*∗*^	4.38 ± 0.35 (11.21)^*∗∗*^	3.71 ± 0.48 (9.74)^*∗*^
EFHB	200	2.27 ± 0.21	6.87 ± 0.56 (3.83)^*∗*^	8.73 ± 0.54 (12.73)^*∗*^	6.94 ± 0.39 (12.73)^*∗*^	5.05 ± 0.31 (17.24)^*∗∗*^	4.52 ± 0.35 (9.35)^*∗*^
EFHB	400	2.70 ± 0.22	8.85 ± 0.41 (8.60)^*∗∗*^	9.83 ± 0.43 (18.16)^*∗∗*^	8.06 ± 0.30 (18.16)^*∗∗*^	6.25 ± 0.29 (19.30)^*∗∗*^	5.16 ± 0.42 (12.26)^*∗*^
NLX	2	2.91 ± 0.32	2.80 ± 0.21	2.76 ± 0.28	2.82 ± 0.21	2.98 ± 0.26	2.70 ± 0.23
NLX + morphine	2 + 5	2.63 ± 0.24	3.95 ± 0.26 (−2.02)^a^	3.81 ± 0.21 (−0.72)^a^	3.95 ± 0.27 (−0.72)^a^	3.91 ± 0.22 (−3.64)^a^	3.62 ± 0.37 (−0.63)^a^
NLX + EFHB	2 + 100	3.05 ± 0.28	3.46 ± 0.34 (−3.42)	3.20 ± 0.35 (−1.35)^b^	3.50 ± 0.18 (−1.35)^b^	2.95 ± 0.28 (−1.07)^b^	2.69 ± 0.41 (−0.04)^b^
NLX + EFHB	2 + 200	2.98 ± 0.24	3.65 ± 0.28 (1.11)^c^	3.71 ± 0.23 (1.21)^c^	3.75 ± 0.24 (1.21)^c^	3.24 ± 0.49 (−0.27)^c^	3.01 ± 0.38 (−0.99)^c^
NLX + EFHB	2 + 400	2.81 ± 0.18	4.02 ± 0.25 (2.07)	4.96 ± 0.39 (2.44)^d^	5.11 ± 0.36 (2.44)^d^	3.75 ± 0.37 (1.28)^d^	3.11 ± 0.27 (−3.49)^d^

Here, each value is presented as the mean ± SEM (*n* = 5). EFHB = ethanol extract of *Ficus hispida*. NLX = naloxone, MPE = Maximum Possible Effect.

^*∗*^Indicating *p* < 0.05 compared with control group (Dunnett's test).

^*∗∗*^Indicating *p* < 0.01 compared with control group (Dunnett's test).

^a^Indicating *p* < 0.001 compared with morphine group (Bonferroni's test).

^b^Indicating *p* < 0.05 compared with morphine group (Bonferroni's test).

^c^Indicating *p* < 0.05 compared with morphine group (Bonferroni's test).

^d^Indicating *p* < 0.01 compared with morphine group (Bonferroni's test).

**Table 6:** Effects of EFHB on carrageenan-induced oedema paw volume in Wistar rats.

Treatment	Dose (mg/kg)	Right hind paw volume (% inhibition)				
		1 h	2 h	3 h	4 h	5 h
Vehicle	10 (ml/kg)	1.03 ± 0.11	1.27 ± 0.16	1.38 ± 0.16	1.44 ± 0.19	1.58 ± 0.12
Indomethacin	10	0.48 ± 0.16 (53.22%)^*∗∗*^	0.54 ± 0.19 (57.80%)^*∗*^	0.54 ± 0.13 (60.76%)^*∗∗*^	0.56 ± 0.18 (61.33%)^*∗*^	0.51 ± 0.18 (67.72%)^*∗∗*^
EFHB	100	0.82 ± 0.14 (19.69%)^*∗*^	0.94 ± 0.14 (26.30%)^*∗*^	0.98 ± 0.19 (29.07%)^*∗*^	0.97 ± 0.15 (32.64%)^*∗*^	1.02 ± 0.16 (35.44%)^*∗*^
EFHB	200	0.76 ± 0.10 (25.54%)^*∗*^	0.78 ± 0.22 (38.90%)^*∗*^	0.84 ± 0.14 (39.24%)^*∗*^	0.85 ± 0.11 (40.97%)^*∗∗*^	0.87 ± 0.17 (44.94%)^*∗∗*^
EFHB	400	0.55 ± 0.17 (46.60%)^*∗*^	0.62 ± 0.18 (51.18%)^*∗∗*^	0.66 ± 0.17 (52.17%)^*∗*^	0.65 ± 0.21 (54.86%)^*∗∗*^	0.64 ± 0.11 (59.49%)^*∗∗*^

Here, each value is presented as the mean ± SEM (*n* = 5). EFHB = ethanol extract of *Ficus hispida*.

^*∗*^Indicating *p* < 0.05 compared with control group (Dunnett's test).

^*∗∗*^Indicating *p* < 0.01 compared with control group (Dunnett's test).

**Table 7:** Effect of EFHB on histamine-induced oedema paw volume in Wistar rats.

Treatment	Dose (mg/kg)	Right hind paw volume (% inhibition)				
		1 h	2 h	3 h	4 h	5 h
Vehicle	10 (ml/kg)	1.15 ± 0.29	1.35 ± 0.26	1.46 ± 0.21	1.57 ± 0.21	1.68 ± 0.24
Indomethacin	10	0.58 ± 0.27 (49.39%)^*∗*^	0.61 ± 0.18 (54.37%)^*∗∗*^	0.62 ± 0.14 (57.42%)^*∗∗*^	0.56 ± 0.11 (64.33%)^*∗∗*^	0.51 ± 0.11 (69.64%)^*∗∗*^
EFHB	100	0.82 ± 0.19 (28.10%)^*∗*^	1.02 ± 0.13 (24.65%)^*∗*^	0.98 ± 0.16 (32.97%)^*∗*^	1.03 ± 0.18 (34.39%)^*∗*^	1.07 ± 0.12 (36.31%)^*∗*^
EFHB	200	0.80 ± 0.17 (29.84%)^*∗*^	0.82 ± 0.15 (39.56%)^*∗*^	0.88 ± 0.21 (39.84%)^*∗*^	0.91 ± 0.15 (42.03%)^*∗*^	0.90 ± 0.16 (46.43%)^*∗*^
EFHB	400	0.60 ± 0.14 (47.99%)^*∗*^	0.68 ± 0.17 (49.63%)^*∗∗*^	0.69 ± 0.15 (52.74%)^*∗∗*^	0.68 ± 0.21 (56.68%)^*∗∗*^	0.67 ± 0.13 (60.12%)^*∗∗*^

Here, each value is presented as the mean ± SEM (*n* = 5). EFHB = ethanol extract of *Ficus hispida*.

^*∗*^Indicating *p* < 0.05 compared with control group (Dunnett's test).

^*∗∗*^Indicating *p* < 0.01 compared with control group (Dunnett's test).

**Table 8:** Effect of EFHB on pentobarbital-induced hypnosis in mice.

Treatment	Dose (mg/kg)	Time of onset of sleep (min)	Total sleeping time (min)
Vehicle	10 (ml/kg)	16.50 ± 0.81	37.80 ± 1.13
Diazepam	1 (i.p.)	4.30 ± 0.16^*∗∗*^	73.50 ± 2.20^*∗∗*^
EFHB	100	9.70 ± 0.57^*∗∗*^	47.90 ± 1.56^*∗*^
EFHB	200	8.20 ± 0.71^*∗∗*^	53.70 ± 1.77^*∗*^
EFHB	400	7.60 ± 0.32^*∗∗*^	60.80 ± 2.01^*∗∗*^

Here, each value is presented as the mean ± SEM (*n* = 5). EFHB = ethanol extract of *Ficus hispida*.

^*∗*^Indicating *p* < 0.01 compared with control group (Dunnett's test).

^*∗∗*^Indicating *p* < 0.001 compared with control group (Dunnett's test).

**Table 9:** Effects of EFHB on open field test in mice.

Treatment	Dose (mg/kg)	Number of movements						
		0 min	30 min	60 min	90 min	120 min	180 min	240 min
Vehicle	10 (ml/kg)	112.30 ± 2.60	109.80 ± 2.15	110.40 ± 1.37	105.50 ± 1.31	101.80 ± 1.55	104.40 ± 0.95	106.30 ± 1.28
Diazepam	1 (i.p.)	101.20 ± 1.09^*∗∗*^	40.50 ± 1.31^*∗∗*^	25.90 ± 1.2^*∗∗*^	15.40 ± 1.5^*∗∗*^	8.90 ± 1.80^*∗*^	7.60 ± 1.48^*∗*^	5.90 ± 1.13^*∗∗*^
EFHB	100	110.50 ± 1.58^*∗*^	78.90 ± 2.06^*∗*^	65.80 ± 1.28^*∗*^	49.60 ± 3.76^*∗*^	43.60 ± 1.9^*∗∗*^	40.20 ± 1.97^*∗*^	37.50 ± 0.82^*∗*^
EFHB	200	108.70 ± 2.01^*∗*^	73.10 ± 2.59^*∗*^	53.60 ± 1.21^*∗*^	45.50 ± 2.31^*∗*^	39.40 ± 2.59^*∗*^	33.80 ± 1.7^*∗∗*^	30.40 ± 0.66^*∗*^
EFHB	400	105.50 ± 2.43^*∗*^	71.20 ± 2.73^*∗∗*^	50.20 ± 2.1^*∗∗*^	39.40 ± 2.4^*∗∗*^	32.80 ± 2.3^*∗∗*^	25.70 ± 1.72^*∗*^	21.70 ± 1.51^*∗∗*^

Here, each value is presented as the mean ± SEM (*n* = 7). EFHB = Ethanolic extract of Ficus hispida.

^*∗*^Indicate *p* < 0.01 compared with control group (Dunnett's test).

^*∗∗*^Indicate *p* < 0.001 compared with control group (Dunnett's test).

**Table 10:** Effects of EFHB on hole cross test in mice.

Treatment	Dose (mg/kg)	Number of movements						
		0 min	30 min	60 min	90 min	120 min	180 min	240 min
Vehicle	10 (ml/kg)	11.10 ± 0.51	10.30 ± 0.68	10.20 ± 0.77	9.70 ± 0.34	9.20 ± 0.36	8.70 ± 0.57	7.50 ± 0.61
Diazepam	1 (i.p.)	10.20 ± 0.98^*∗∗*^	4.20 ± 0.6^*∗∗*^	2.10 ± 0.33^*∗∗*^	1.50 ± 0.15^*∗∗*^	0.90 ± 0.11^*∗∗*^	0.50 ± 0.12^*∗∗*^	0.30 ± 0.08^*∗∗*^
EFHB	100	11.20 ± 0.37^*∗*^	7.50 ± 0.55^*∗*^	7.30 ± 0.65^*∗*^	7.10 ± 0.80^*∗*^	6.80 ± 0.50^*∗*^	6.40 ± 0.25^*∗*^	6.10 ± 0.07^*∗*^
EFHB	200	12.30 ± 0.18^*∗∗*^	7.10 ± 0.43^*∗*^	6.90 ± 0.53^*∗*^	6.50 ± 0.79^*∗*^	6.10 ± 0.58^*∗*^	5.80 ± 0.66^*∗*^	5.20 ± 0.35^*∗*^
EFHB	400	11.40 ± 0.90^*∗*^	6.80 ± 0.36^*∗∗*^	6.30 ± 0.40^*∗*^	5.90 ± 0.89^*∗*^	5.30 ± 0.57^*∗*^	4.90 ± 0.64^*∗*^	4.30 ± 0.31^*∗∗*^

Here, each value is presented as the mean ± SEM (*n* = 7). EFHB = ethanolic extract of *Ficus hispida*.

^*∗*^Indicating *p* < 0.01 compared with control group (Dunnett's test).

^*∗∗*^Indicating *p* < 0.001 compared with control group (Dunnett's test).
